# TALEN-mediated functional correction of human iPSC-derived macrophages in context of hereditary pulmonary alveolar proteinosis

**DOI:** 10.1038/s41598-017-14566-8

**Published:** 2017-11-09

**Authors:** Alexandra Kuhn, Mania Ackermann, Claudio Mussolino, Toni Cathomen, Nico Lachmann, Thomas Moritz

**Affiliations:** 1 Institute of Experimental Hematology, Hannover Medical School, Hannover, Germany; 2 RG Reprogramming and Gene Therapy, REBIRTH Cluster of Excellence, Hannover, Germany; 3 JRG Translational Hematology of Congenital Diseases, REBIRTH Cluster of Excellence, Hannover, Germany; 4 Institute for Transfusion Medicine and Gene Therapy, Medical Center - University of Freiburg, Freiburg, Germany; 5 Center for Chronic Immunodeficiency, Medical Center - University of Freiburg, Freiburg, Germany; 6 Faculty of Medicine, University of Freiburg, Freiburg, Germany

## Abstract

Hereditary pulmonary alveolar proteinosis (herPAP) constitutes a rare, life threatening lung disease characterized by the inability of alveolar macrophages to clear the alveolar airspaces from surfactant phospholipids. On a molecular level, the disorder is defined by a defect in the *CSF2RA* gene coding for the GM-CSF receptor alpha-chain (CD116). As therapeutic options are limited, we currently pursue a cell and gene therapy approach aiming for the intrapulmonary transplantation of gene-corrected macrophages derived from herPAP-specific induced pluripotent stem cells (herPAP-iPSC) employing transcriptional activator-like effector nucleases (TALENs). Targeted insertion of a codon-optimized *CSF2RA*-cDNA driven by the hybrid cytomegalovirus (CMV) early enhancer/chicken beta actin (CAG) promoter into the *AAVS1* locus resulted in robust expression of the *CSF2RA* gene in gene-edited herPAP-iPSCs as well as thereof derived macrophages. These macrophages displayed typical morphology, surface phenotype, phagocytic and secretory activity, as well as functional *CSF2RA* expression verified by STAT5 phosphorylation and GM-CSF uptake studies. Thus, our study provides a proof-of-concept, that TALEN-mediated integration of the *CSF2RA* gene into the *AAVS1* safe harbor locus in patient-specific iPSCs represents an efficient strategy to generate functionally corrected monocytes/macrophages, which in the future may serve as a source for an autologous cell-based gene therapy for the treatment of herPAP.

## Introduction

The reprogramming of human somatic cells into induced pluripotent stem cells (iPSCs)^1^ in combination with precise genome engineering technologies employing zinc-finger nucleases (ZFNs), transcription activator-like effector nucleases (TALENs) or RNA-guided nucleases, has rapidly advanced our options for disease modeling, drug screening and cell replacement therapies^2–7^ and has had a marked impact on personalized medicine including gene and cell therapy strategies^8^. Within the lympho-hematopoietic system, designer nuclease-mediated homology-directed repair (HDR) has already been applied for genetic correction of disorders such as chronic granulomatous disease^7,9,10^, ß-hemoglobinopathies^11–13^ and severe combined immunodeficiency (SCID)^14,15^. In this context, the intron 1 of the *PPP1R12C* gene on human chromosome 19, originally described as the favored integration site for adeno-associated viruses and also referred to as *AAVS1* locus, has been exploited as a predefined “safe harbor” integration site allowing for the stable expression of transgenic sequences in pluripotent stem cells (PSCs) and thereof differentiated progeny^7,10,16–18^. We here have targeted the *AAVS1* site to establish a novel cell and gene therapy approach for hereditary pulmonary alveolar proteinosis (herPAP) patients.

HerPAP constitutes a life-threatening, congenital lung disease usually diagnosed in childhood, which is defined by extensive accumulation of lipoprotein material in the alveolar spaces of the lungs resulting in progressive respiratory insufficiency and in severe cases respiratory failure and death (reviewed in^19^). Accountable for the disease are abnormalities in the genes coding for the granulocyte/macrophage colony-stimulating-factor receptor alpha- (*CSF2RA*, CD116) or beta- (*CSF2RB*, CD131) subunit. While the alpha-subunit of the receptor is responsible for low-affinity interaction with human GM-CSF, binding affinity is enhanced and intracellular signal transduction is initiated upon binding to the beta-subunit, which is common with the receptors for human interleukin (IL)-3, -5 and GM-CSF^20^. Defective GM-CSF receptor signaling, in most patients due to defects in *CSF2RA*, results in abnormal development and functionality of alveolar macrophages (AM) and an inability to maintain pulmonary surfactant homeostasis^21^. Repetitive broncho-alveolar lavage performed under general anesthesia currently provides the only effective therapy. However, this procedure has to be repeated every 4-8 weeks, is associated with considerable cardiopulmonary risks and severely affects the quality of life of herPAP patients. Allogeneic bone marrow transplantation (allo-BMT) or hematopoietic stem cell based gene therapy, both of which have been used successfully for the treatment of other congenital diseases affecting the lympho-hematopoietic compartment^22–24^ have proven problematic in herPAP, as the pre-existing lung damage prevents adequate chemo- and radiotherapeutic preconditioning^25^. Recently, however, pulmonary macrophage transplantation has been described as a highly effective therapeutic approach for herPAP in two murine disease models^26,27^.

Given this background we aimed to develop a gene therapy approach for herPAP using iPSC and TALEN technology to correct the *CSF2RA*-mediated form of the disease. We here describe for the first time the TALEN-mediated genetic integration of a codon-optimized *CSF2RA* transgene (*CSF2RA* ^*coop*^) into the *AAVS1* locus of herPAP patient-derived iPSCs restoring GM-CSF receptor functionality and correcting the *in vitro* disease phenotype of herPAP iPSC-derived monocytes/macrophages.

## Results

### Targeted insertion of the *CSF2RA*^*coop*^ gene into the AAVS1 safe harbor locus by homologous recombination using TALENs and herPAP patient-derived iPSCs

**Figure 1:**
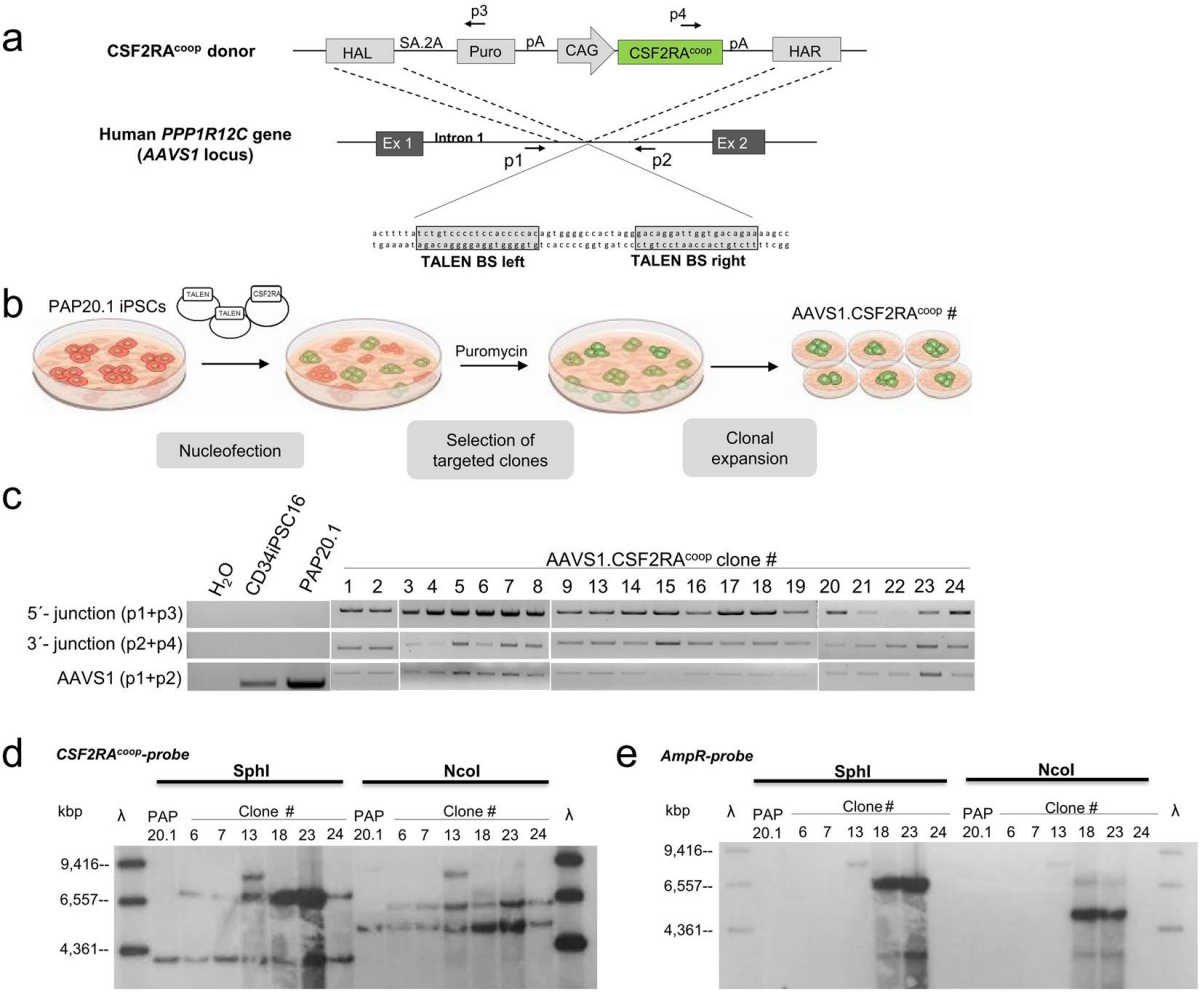
Generation and genotyping of gene edited PAP patient-derived iPSCs. (**a**) Scheme of the *AAVS1* target site and the CSF2RA^coop^ donor plasmid. The donor plasmid is used as template for homologous recombination at the intended target site. The puromycin selection cassette and the CAG-driven *CSF2RA* ^*coop*^ gene are flanked by *AAVS1*-specific homology arms. The *AAVS1*-specifc TALEN binding sites are located in intron 1 of the *PPP1R12C* gene (*AAVS1* locus). (**b**) Experimental scheme for the generation, selection and expansion of clones with *CSF2RA* ^*coop*^ integration within the *AAVS1* locus of herPAP iPSCs. (**c**) Verification of site-specific integration of the expression cassette via PCR in 21 puromycin selected *CSF2RA* ^*coop*^ integrated clones. Genomic DNA of the resistant clones was extracted for junction PCR using primers targeting the 3′(p2 + p4) and 5′(p1 + p3) -junction between the genomic site and the donor cassette. Positions of primers are indicated in (a). Site-specific integration was observed in all clones, with mono-allelic integration in 20 clones (95%; *AAVS1*-specific band (p1 + p2) present) and bi-allelic integration in one clone (5%; no *AAVS1*-specific band). The contrast and brightness were slightly adjusted using the Image Lab Software. Images are cropped from different gels (borders of the individual gels are indicated by white spaces) and grouped according to the numbering. Full-length agarose gels are presented in Supplementary Figure S1. (**d**,**e**) Six mono-allelic targeted clones were further analyzed for verification of targeted integration via southern blot. For southern blot analysis genomic DNA of the six selected iPSC clones was blotted onto membrane after digestion with SphI or NcoI and hybridized with either a^32^P-labeled CSF2RA^coop^ probe (873 bp; (**d**)) or ampicillin probe (1095 bp; (**e**)). For all six clones integration of *CSF2RA* ^*coop*^ could be verified (black arrows; 7,1 kb, SphI; 6,3 kb, NcoI; see (**d**)). Additionally, random integration of the donor in clones #13, #18 and #23 was monitored using an ampicillin probe. Full-length southern blots are presented in Supplementary Figure S3. HAL, homology arm left; HAR, homology arm right; SA, splice acceptor; 2 A, self-cleaving peptide; Puro, puromycin resistance gene; CAG, CMV early enhancer/chicken ß-actin promoter; pA, poly A site; BS, binding site.

Programmable nucleases such as TALENs represent a promising technology to introduce DNA double-strand breaks and allow for a homology-directed repair process precisely targeting the gene of interest into a pre-defined genomic locus. We here adapted this technology to develop a cell and gene therapy approach for herPAP based on macrophages derived from gene corrected PAP-disease specific iPSCs. IPSCs were derived from a herPAP patient diagnosed with a pre-mature stop codon in exon 7 of the *CSF2RA* gene and generation as well as characterization of the iPSC clone (PAP20.1) has been described previously^28^. As mutations leading to herPAP can be distributed throughout the *CSF2RA* gene which results in PAP-phenotypes of varying severity^21^, we here evaluated the feasibility of a targeted integration of the codon-optimized version of the *CSF2RA* transgene (*CSF2RA* ^*coop*^) into the *PPP1R12C* gene intron 1, better known as *AAVS1* locus, as a comprehensive therapeutic strategy. For this purpose, we used a gene-trap donor construct, expressing a puromycin resistance gene from the endogenous *PPP1R12C* promoter^7^ to easily select for targeted iPSC clones (Fig. 1a,b). The donor construct, which also contained a codon-optimized *CSF2RA*-cDNA (*CSF2RA* ^*coop*^) driven by the CMV early enhancer/chicken beta actin (CAG) promoter^16,29,30^ was targeted to the *AAVS1* safe harbor locus by homology-directed repair using *AAVS1*-specific TALENs that were previously validated for their on-target activity and specificity^31^. These TALENs and the donor construct were delivered by nucleofection into PAP20.1-iPSCs, achieving a transfection efficiency of approximately 35% (data not shown). Out of 24 clones, only 21 survived the initial passaging, and we observed a targeting efficiency of 100% after puromycin selection with mono-allelic targeting of the AAVS1.CSF2RA^coop^ donor cassette in 20 out of 21 (95%) selected iPSC clones and one clone (i.e. clone #15) showing bi-allelic targeting as demonstrated by PCR (Fig. 1c, Supplementary Fig. S1). To confirm site-specific integration into the *AAVS1* safe harbor, a representative Sanger sequencing of the junctions between the genomic locus and the insert in the targeted iPSC clone #6 was performed (Supplementary Fig. S2). For further studies six mono-allelic targeted clones were selected and 50% of these (#6, #7, #24) showed a single integration of the *CSF2RA* ^*coop*^ transgene within the *AAVS1* locus without additional random integration of the donor backbone using southern blotting and ^32^P-labeled probes specific for the *CSF2RA* ^*coop*^ and the ampicillin resistance transgenes respectively (Fig. 1d,e and Supplementary Fig. S3). These results confirm the on-target integration of the therapeutic *CSF2RA* ^*coop*^ transgene within the *AAVS1* genomic locus in our PAP-patient-specific iPSCs. Two of these *CSF2RA* ^*coop*^-targeted clones (#6 and #7) were chosen for further analysis.

### Characterization of AAVS1.CSF2RA^coop^ targeted iPSCs

**Figure 2:**
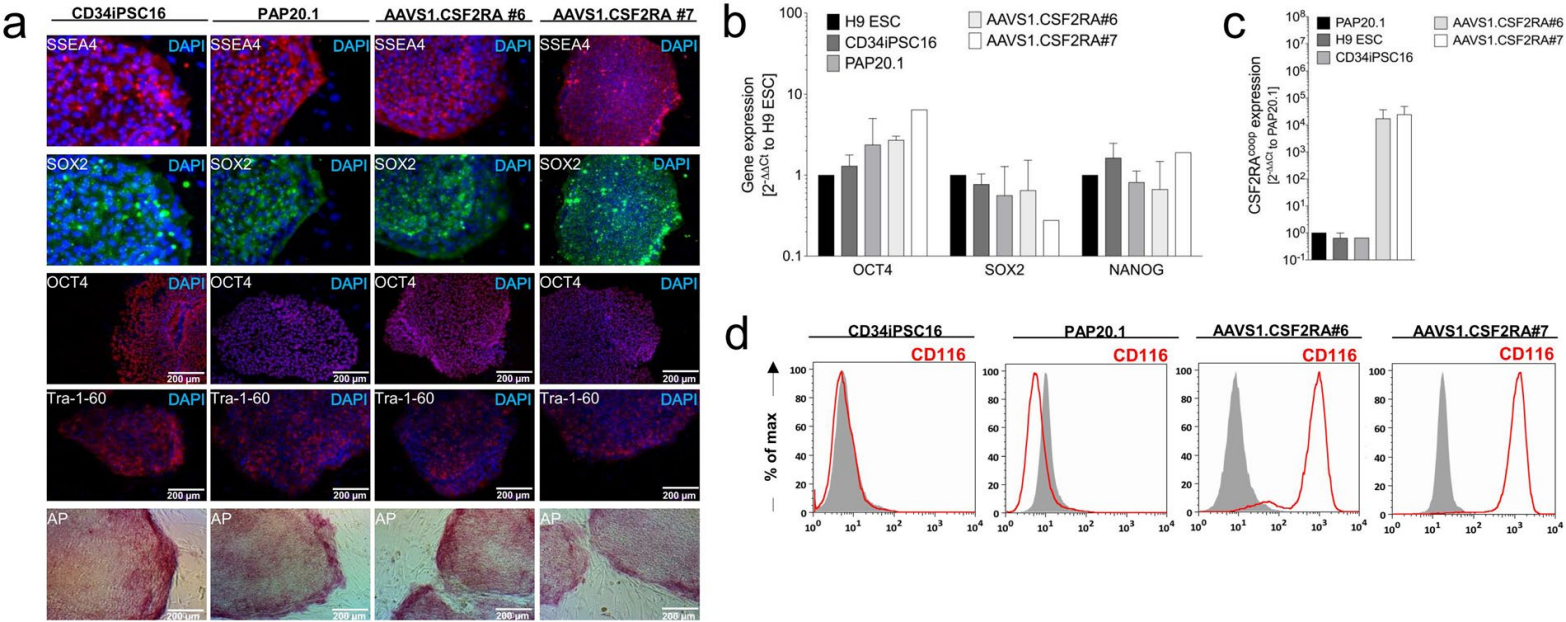
Characterization of AAVS1.CSF2RA^coop^ iPSCs. **(a)** Immunohistochemical characterization of iPSCs. Healthy donor-derived hCD34iPSC16, PAP patient-derived iPSC20.1, as well as AAVS1.CSF2RA^coop^ clones #6 and #7 express typical pluripotency-related surface markers and transcription factors including *SSEA4, SOX2, OCT4, Tra-1-60* and display alkaline phosphatase activity. **(b)** qRT-PCR-based gene expression analysis of pluripotency-related transcription factors relative to human *GAPDH* (housekeeping gene) and H9 ESCs used as reference. The transcription factors *OCT4, SOX2* and *NANOG* are expressed by CD34iPSC16, the PAP20.1 as well as AAVS1.CSF2RA^coop^ #6 and #7. (H9, n = 3; CD34iPSC16, n = 3; PAP20.1, n = 3; #6, n = 2; #7, n = 2) **(c)** Gene expression analysis of *CSF2RA* ^*coop*^. PAP20.1 was used as negative control iPSC line to verify *CSF2RA* ^*coop*^ expression in the edited AAVS1.CSF2RA^coop^ #6 and #7. (H9, n = 3; CD34iPSC16, n = 2; PAP20.1, n = 3; #6, n = 3; #7, n = 3) **(d)** Histograms depicting GM-CSF receptor (CD116) expression on iPSCs by flow cytometry. Mouse IgG1κ isotype controls (solid gray), CD116 (red); H9 ESC, H9 embryonic stem cells; DAPI, 4′,6-diamidino-2-phenylindole.

To further characterize these clones, the expression of pluripotency-related surface markers and transcription factors was determined by immunohistochemistry and/or quantitative RT-PCR analysis. Both clones showed *SSEA4, SOX2, OCT4, Tra-1-60* and*NANOG* expression as well as alkaline phosphatase activity (Fig. 2a,b). Furthermore, *CSF2RA* ^*coop*^ expression in AAVS1.CSF2RA^coop^ iPSC clones #6 and #7 was verified by qRT-PCR and flow cytometric analysis whereas CD116 was absent in non-corrected parental PAP20.1 iPSCs (Fig. 2c,d). Thus, our gene-edited iPSC clones retained their pluripotency after targeted integration while the CAG promoter allowed for effective expression of the *CSF2RA* ^*coop*^ transgene from the *AAVS1* safe harbor site.

### Generation of monocytes/macrophages from genetically corrected iPSCs

**Figure 3:**
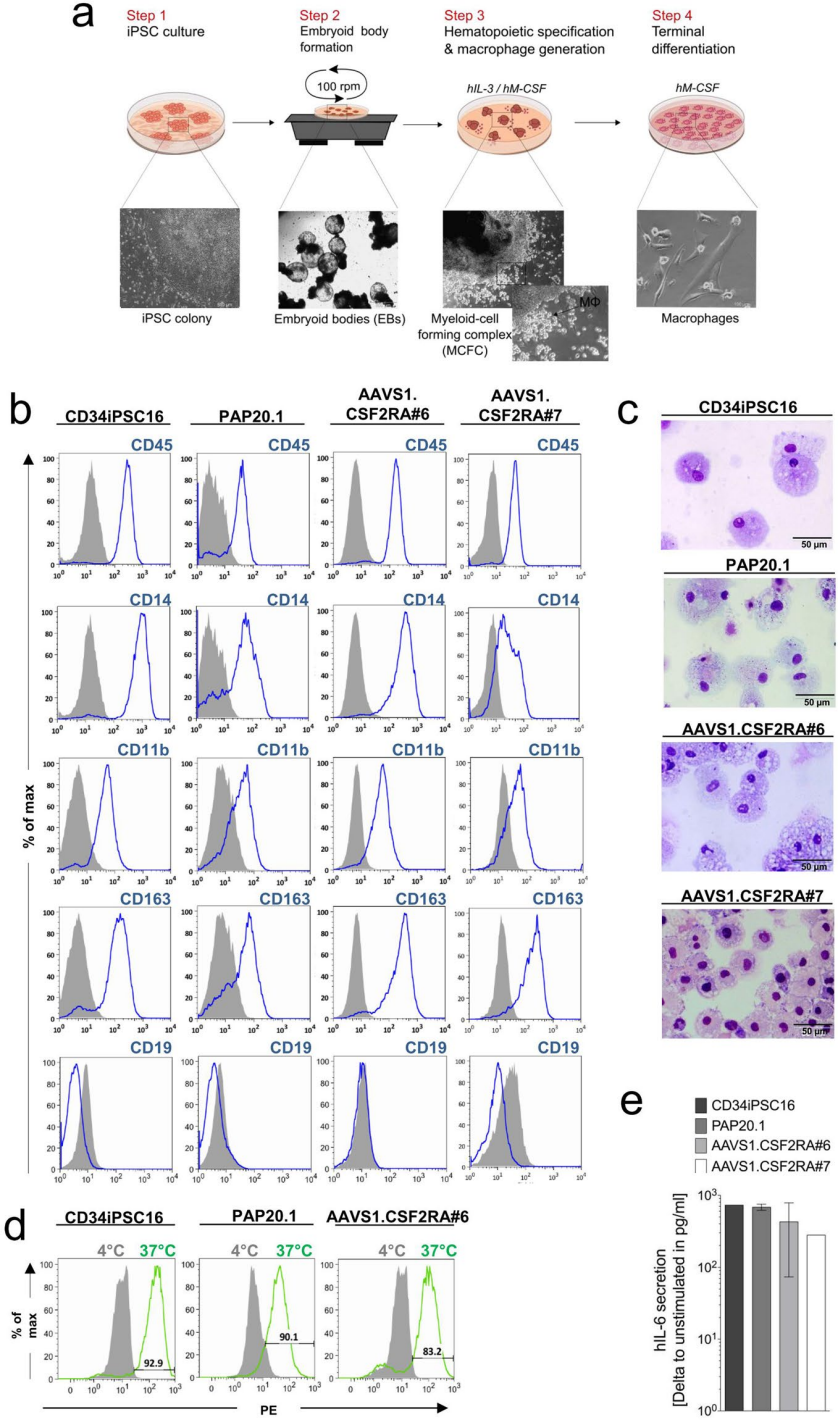
Generation and characterization of iPSC-derived monocytes/macrophages. (**a**) Schematic overview of the hematopoietic differentiation protocol for the generation of monocytes/macrophages from human iPSC. (**b**) Flow cytometric analysis of monocytes/macrophages from the differentiation culture. Histograms show surface marker expression (solid gray = isotype controls/unstained cells, blue = surface marker). (**c**) Evaluation of cellular morphology via cytospin analysis (scale bar 50 µm). (**d**) Monocytes/macrophages are able to phagocytose pH-sensitive fluorochrome-labeled pHrodo E.*coli* and (**e**) upon LPS stimulation secrete human IL-6 analyzed by ELISA (CD34iPSC16, n = 2; PAP20.1, n = 4; #6, n = 3; #7, n = 2).

We next determined whether expression of the *CSF2RA* ^*coop*^ transgene from the *AAVS1* safe harbor locus was sufficient to restore the GM-CSF receptor alpha-chain expression in monocytes and macrophages derived from gene-edited herPAP iPSCs *in vitro*. For these studies, the AAVS1.CSF2RA^coop^ clones #6 and #7 as well as the parental PAP20.1 iPSC clone and healthy donor-derived CD34iPSC16 iPSCs were differentiated into monocytes/macrophages, utilizing a previously reported embryoid body (EB)-based differentiation protocol to generate mature hCD14^+^hCD11b^+^ cells^32^ (Fig. 3a). Flow cytometry analysis demonstrated a classical macrophage surface marker profile hCD45^+^hCD14^+^hCD11b^+^hCD163^+^hCD19^−^ for CD34iPSC16-, PAP20.1- and AAVS1.CSF2RA#6- and #7-derived macrophages (Fig. 3b) and classical macrophage morphology on May-Grünwald-Giemsa stained cytospins (Fig. 3c). When CD34iPSC16-, PAP20.1- and AAVS1.CSF2RA#6-derived macrophages were investigated in a fluorescence-labeled pHrodo E.*coli*-based phagocytosis assay, macrophages derived from all clones showed profound bacterial uptake capacities at 37 °C (Fig. 3d). Moreover, all macrophages including those derived from AAVS1.CSF2RA #7 secreted substantial levels of human Interleukin-6 (hIL-6) upon exposure to bacterial LPS (Fig. 3e). Thus, *CSF2RA* ^*coop*^ gene-edited PAP-iPSCs allowed for efficient macrophage differentiation giving rise to cells displaying surface phenotypes, morphologies as well as phagocytic and secretory properties indistinguishable from control macrophages differentiated from healthy donor-derived CD34iPSC16.

### Functional evaluation of the GM-CSF receptor alpha-chain in genetically corrected iPSC-derived monocytes/macrophages

**Figure 4:**
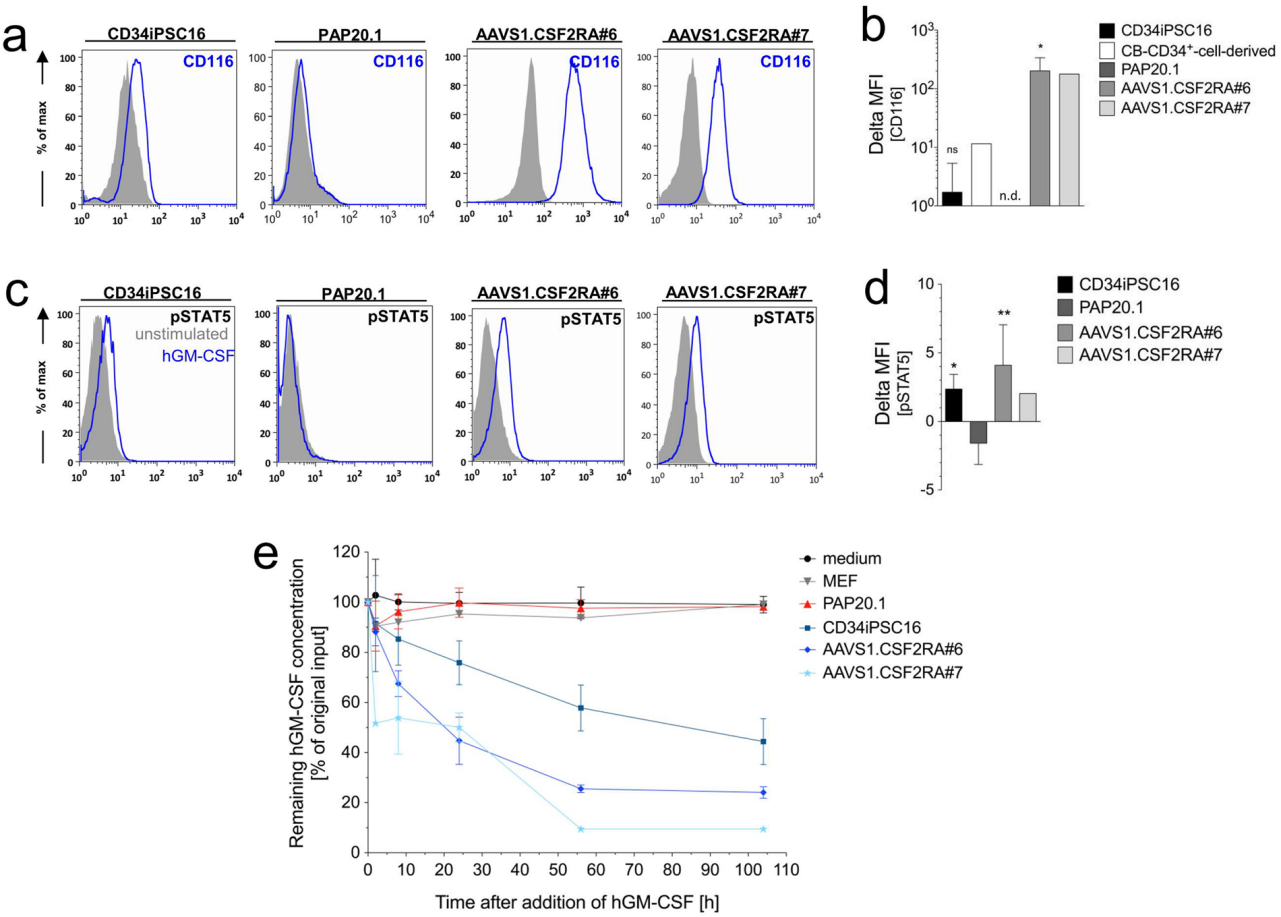
Functional evaluation of the GM-CSF receptor on gene edited iPSC-derived monocytes/macrophages. (**a**) Flow cytometric analysis of *CSF2RA* (CD116) expression on freshly harvested iPSC-derived monocytes/macrophages (solid gray = respective isotype control, blue = CD116) and (**b**) summary of delta MFIs (ΔMFI = MFI CD116 - MFI isotype) values (CD34^+^ cord blood cell-derived macrophages, n = 1; CD34iPSC16, n = 4; PAP20.1, n = 3; AAVS1.CSF2RA^coop^#6, n = 4; AAVS1.CSF2RA^coop^#7, n = 2; mean (SD)). (**c**) GM-CSF induced STAT-5 phosphorylation (pSTAT5) evaluated via a flow cytometry-based assay (solid gray line = unstimulated control, blue line = hGM-CSF stimulated cells) and **d)** summary of corresponding delta MFI values (CD34iPSC16, n = 6; PAP20.1, n = 3; AAVS1.CSF2RA^coop^#6, n = 5; AAVS1.CSF2RA^coop^#7, n = 2; mean (SD)). (**e**) GM-CSF clearance following incubation in 1 ng/ml hGM-CSF-supplemented medium as analyzed by hGM-CSF ELISA. */**Gives significance of p < 0.05/0.01 by one-way ANOVA compared to PAP20.1; ns, not significant; n.d., not detected.

Due to mutations in the *CSF2RA* gene, macrophages derived from PAP patients fail to express the GM-CSF receptor alpha-chain, leading to distinct functional insufficiencies of these cells^33^. To demonstrate the presence and functionality of the GM-CSF receptor alpha-chain in *CSF2RA* ^*coop*^ gene-edited iPSC-derived macrophages, we first evaluated CD116 expression by flow cytometry. While for the non-corrected, PAP20.1- derived macrophages no CD116 expression was observed, macrophages derived from control CD34iPSC16 and gene-targeted AAVS1.CSF2RA#6 or #7 clones displayed high CD116 expression on their surface (Fig. 4a). However, CD116 expression was clearly more pronounced on gene-targeted AAVS1.CSF2RA-derived macrophages than the physiological expression levels on CD34iPSC16- or cord blood-derived monocytes/macrophages (Fig. 4b). To evaluate the functional correction of genetically modified AAVS1.CSF2RA-derived macrophages we next analyzed GM-CSF-induced phosphorylation of the signal transducer and activator of transcription 5 (STAT5), a major downstream effector of GM-CSF receptor signaling^28,34^. When GM-CSF induced STAT-5 phosphorylation was analyzed via a flow cytometry based assay, PAP20.1 iPSC-derived macrophages were unable to phosphorylate STAT5 (pSTAT5), whereas profound pSTAT5 levels were induced in AAVS1.CSF2RA^coop^ #6- and #7- derived macrophages (Fig. 4c,d). As another GM-CSF receptor dependent function, the ability of the *AAVS1*-targeted iPSC-derived macrophages to take up and clear GM-CSF from the supernatant was determined by ELISA. While in control settings (medium only, murine embryonic fibroblasts and PAP20.1-derived macrophages) GM-CSF levels in the medium remained constant, macrophages derived from CD34iPSC16 controls as well as both *AAVS1*-targeted clones significantly reduced GM-CSF in the medium over time (Fig. 4e). Thus, our data demonstrate that not only robust CD116 expression levels in macrophages can be achieved by targeting the codon-optimized *CSF2RA* gene into the *AAVS1* safe harbor site of PAP patient-derived iPSCs but also receptor downstream signaling and GM-CSF receptor dependent functions such as GM-CSF clearance could be restored.

## Discussion

Cell and gene therapy employing patient-derived iPSCs holds great potential to treat rare hematologic diseases, such as herPAP, for which new therapeutic treatments are urgently required. Current treatment options to clear the lungs of herPAP patients from surplus surfactant material are based on a repetitive and invasive broncho-alveolar lavage procedure entailing high risks for the patient. As demonstrated here, the reprogramming of fibroblasts or peripheral blood cells into patient-specific iPSCs followed by their genetic correction and differentiation towards macrophages represents a suitable approach to generate a donor source for cell and gene therapy in herPAP. HerPAP specific human as well as murine iPSCs, which upon macrophage differentiation recapitulate the herPAP phenotype, have been described before^28,34,35^ and for human herPAP-iPSCs even the successful lentiviral delivery of a *CSF2RA* cDNA followed by the subsequent *in vitro* differentiation into monocytes/macrophages with restored GM-CSF dependent functionality has been shown^28,34^. However, lentiviral integration into the genome is a semi-random process accompanied by risks such as insertional mutagenesis and variegation or even silencing of transgene expression^36–38^. Insertional mutagenesis, at least in the lympho-hematopoietic system, can be expected to be less of a problem with the transplantation of macrophages, as it is observed primarily in the stem and early progenitor cell compartment rather than more differentiated myeloid or lymphoid cells^39,40^. Retroviral transgene silencing, on the other hand, has been described extensively for pluripotent cells and particularly during the early differentiation steps of these cells^41–43^.

On this background precise genome engineering technologies, such as TALENs, allowing for the site-specific correction of distinct mutations or the insertion of defined genomic sequences within the genome of iPSCs and their differentiated progeny appear highly attractive. Recently, TALENs were demonstrated to correct the disease phenotype in SCID-X1-iPSCs and thereof derived mature cells^44^, similar to what was achieved for the correction of ß-Thalassemia iPSCs and differentiated erythroblasts^13^. With the emergence of RNA-guided nucleases, CRISPR/Cas9-mediated correction of congenital lympho-hematopoietic diseases were reported^9,45–47^. Several of these studies targeted the gene of interest into the well-characterized *AAVS1* safe harbor site^3,7,16,48^. This included approaches to functionally correct granulocytes derived from X-CGD iPSCs employing the same TALEN pair as utilized here to direct site specificity of the integration^7,31^. So far, studies on the activity, toxicity and specificity of these *AAVS1*-specific TALENs revealed marginal cytotoxic and off-target effects, while showing good on-target activity^7,31^. Thus, to optimize transgene expression, the *AAVS1* site was chosen as the target for our transgene cassette.

Although the *AAVS1* site is widely regarded as a safe harbor site supporting robust, non-variegated transgene expression, transgene silencing within the *AAVS1* locus associated with DNA methylation has been described^49^. As a safeguard we thus utilized the strong ubiquitously active CAG promoter to enable stable and reproducible expression of our therapeutic transgene from the *AAVS1* locus, as recently shown in an similar strategy to stably express a green fluorescent protein in cells differentiated from human iPSCs^16^. In our hands, this design already allowed for high and sustained expression of the *CSF2RA* ^*coop*^ transgene from a mono-allelic copy of the expression cassette in pluripotent and myeloid cells without signs of silencing during the differentiation process. Should variegation of transgene expression during the differentiation process occur in the future, epigenetic regulatory elements including ubiquitous chromatin opening elements (UCOEs) may be incorporated into the expression constructs to promote an even stronger anti-silencing environment^37,43,48,50^. Likewise, if the constitutive expression of *CSF2RA* from the CAG promoter turns out problematic e.g. in pluripotent cells or early precursors, transgene expression may be restricted to the myeloid lineage utilizing myeloid-specific promoters, including the MRP8^51^ or miR223^52^ promoter. However, a more defined assessment of this topic clearly will require *in vivo* transplantation experiments.

To circumvent these problems and facilitate so called “physiologic” transgene expression under control of the endogenous promoter region, integration of transgenes or parts thereof directly into the deficient gene locus has been demonstrated^6,44,53^. In *CSF2RA* deficiency, physiologic regulation might be achieved by the introduction of a “super exon” within the endogenous *CSF2RA* locus. Here, introduction of the *CSF2RA* -cDNA in proximity to the transcriptional start of the wild type gene would address all different mutations spread over the *CSF2RA* locus^21^ while expression of the malfunctioned wild type gene is omitted. An alternative to achieve physiological expression from the endogenous control elements is the mutation-specific correction of the defective gene sequences. In this line, Zou *et al*. have provided a paradigmatic strategy for a gene therapy of monogenic disorders of the hematopoietic system by the site-specific correction of the sickle cell point mutation within patient-specific iPSCs^6^. These data demonstrated expression of 25–40% of the ß-globin gene in differentiated erythrocytes^6^. In our PAP20.1-iPSCs this approach will require repair of the pre-mature stop-codon mutation at position 199 of the *CSF2RA* gene. However, in the overall population of herPAP patients this strategy will turn out quite cumbersome given the magnitude of different mutations described in the *CSF2RA* gene already^21^.

All these strategies depend on the efficient differentiation of the gene edited PAP-iPSCs into mature and functional monocyte/macrophages. While a number of protocols have been used successfully to generate myeloid cells from iPSCs^32,54,55^ the protocol applied here yielded highly efficient and reproducible generation of functional macrophages displaying a typical surface marker profile and morphology. Interestingly, minor modifications in the protocol also allow for the efficient generation of other hematopoietic cell types such as granulocytes, dendritic cells or erythrocytes^32^ (own unpublished data). Moreover, recently the *in vitro* generation of cells with a distinct alveolar macrophage-like phenotype and gene expression pattern from murine iPSCs has been described and pulmonary transplantation of such AM-like cells yielded effective engraftment and amelioration of the pulmonary disease phenotype in ADA^(−/−)^ mice^56^. Along the same line, we have observed marked improvement of herPAP associated alveolar proteinosis following the intratracheal transplantation of healthy, iPSC-derived murine or human macrophages in two murine *in vivo* disease models similar to the effects observed previously with bone marrow-derived macrophages^26,27^ (manuscript submitted).

In summary, we here report that genetic targeting of a therapeutic cassette expressing the *CSF2RA* ^*coop*^ transgene from the ubiquitous CAG promoter into the *AAVS1* locus corrects the disease phenotype in herPAP-specific iPSCs and their progeny. In our *in vitro* model, targeted iPSCs and thereof derived macrophages showed stable *CSF2RA* expression and restored GM-CSF dependent signaling and functionality such as STAT5 phosphorylation or GM-CSF uptake. Certainly, extensive further studies particularly in animal models will be required to underline the feasibility, safety and efficacy of gene corrected iPSC-derived macrophages to be used as a potential therapy for herPAP. Nevertheless, even the current data support the concept that genetic targeting strategies in combination with efficient differentiation protocols will provide a powerful tool for autologous iPSC-based therapies in the future.

## Experimental Procedures

### Study Subject

This study was approved by the institutional review board of the Hannover Medical School, Hannover, Germany. All subjects or their legal guardians gave written informed consent; minors gave assent. In addition to this, all methods were performed in accordance with the relevant guidelines and regulations. For material information see^28^.

### Plasmids and donors

The *AAVS1* locus-specific TALEN expression plasmids were previously described in^31^. The AAVS1.CSF2RA^coop^ donor was constructed using standard cloning technologies. The plasmid contains homology arms of ~750 bp flanking the puromycin resistance gene, a splice acceptor site (SA), a self-cleaving peptide sequence (2A), a BGH poly(A), the CAG promoter driving the expression of a codon-optimized *CSF2RA* cDNA^28^ and a HSV-TK Poly(A).

### Cell culture

Human PAP20.1 iPSCs^28^ and healthy CD34iPSC16^28,32,]^ ^58^ were maintained on CF-1 murine embryo fibroblasts (MEF) in Knock Out Dulbecco’s modified Eagle medium (KO-DMEM) supplemented with 20% knock out serum replacement, 1% penicillin/streptomycin, 1% non-essential aminoacids, 1 mmol/l L-Glutamine, 0.2% ß-mercaptoethanol and 10 ng/ml basic fibroblast growth factor (bFGF) (Peprotech, Hamburg, Germany) at 37 °C with 5% CO_2_ incubation. Passaging of the iPSCs was performed every 7–10 days using 2 mg/ml collagenase IV (Invitrogen, Carlsbad, CA, USA). The reagents were obtained from Thermo Fisher Scientific.

### Nucleofection and gene editing of human iPSC

Nucleofection of human PAP 20.1 iPSCs and human CD34iPSC16 was performed using the Nucleofector 2b device (Lonza, Basel, Switzerland) and the Human Stem Cell kit 2 (Lonza, Basel, Switzerland) according to the manufacturer’s instructions. In brief, iPSCs were grown to sub-confluency and disaggregated into single cells using TrypLE. For nucleofection 2 × 10^6^ cells were resuspended in nucleofection solution supplemented with 3.8 µg donor DNA plasmid or 1.2 µg pMax-GFP transfection control and 0.6 µg each TALEN DNA plasmid. Transfection efficiency was determined by flow cytometry 48 h post nucleofection. Correctly targeted cells were selected in standard medium supplemented with 0.5 µg/ml puromycin for 7–10 days, starting 48 h post nucleofection. Positive colonies were picked, further expanded and analysed.

### Genotyping of gene edited iPSCs

Extraction of genomic DNA was performed with the GenElute mammalian genomic DNA miniprep kit (Sigma Aldrich, MO, USA). For targeted integration PCRs Phire Hot Start II Polymerase (Thermo Fisher Scientific, MA, USA) was used under standard conditions. To detect the 5′-junction and 3′-junction of targeted integrated (TI) donor DNA and genomic DNA the following primers were used: 5′-TI_AAVS1_fwd: 5′-CCAGCTCCCATAGCTCAGTCTG-3′, 5′-TI_Puro_rev: 5′-GGTCCTTCGGGCACCTCGAC-3′, 3′-TI_AAVS1_rev: 5′-GGGCTCAGTCTGAAGAGCAGAG-3′, 3′-TI_CSF2RA^coop^_fwd: 5′-GGGCAGCGTGTACATCTACG-3′. To discriminate between clones that were targeted mono-allelic or bi-allelic, the AAVS1_fwd and AAVS1_rev primers were used.

### Electrophoretic gels and blots

For agarose gel imaging the Biorad Molecular Imager Geldoc XR + (Model: Universal Hood II) and the Image Lab Software v3.0 were used, applying an auto exposure time of 0.5 sec using UV trans illumination. Slight adjustments in brightness and the gamma value were performed using the image transform tool from Image Lab Software.

### Southern blot analysis

Standard Southern Blotting techniques were applied. In brief, 10 µg of genomic DNA was digested with either SphI or NcoI (Thermo Fisher Scientific, MA, USA) and separated by standard TAE agarose gel electrophoresis. The digested genomic DNA was transferred onto a Biodyne B nylon membrane (Pall Life Sciences) and analysed with radioactively labeled ^32^P-probes using the DecaLabel^TM^ DNA labeling kit (Thermo Fisher Scientific, MaA, USA). The CSF2RA-probe (873 bp) was generated by digesting the donor with EcoNI and EcoRI (Thermo Fisher Scientific, MA, USA). An additional ampicillin-probe (1059 bp) was generated by PCR amplification and used for re-hybridization after alkaline stripping of the membrane. The CSF2RA-probe will detect a 7139 bp large targeted integration band for genomic DNA digested with SphI and a 6320 bp large targeted integration fragment when digested with NcoI. For the AmpR-probe a random integration band of unknown size will be generated with either digestion profile. HindIII digested P^32^-labeled bacteriophage lambda DNA served as standard.

### Real-time quantitative reverse transcription PCR analysis

For detection of gene expression levels, cells were cultured under standard conditions, harvested and RNA was isolated using phenol chloroform followed by cDNA synthesis using the RevertAid H Minus First Strand cDNA Synthesis Kit (Thermo Fisher Scientific, MA, USA). For determination of *CSF2RA* ^*coop*^ gene expression SYBR Green PCR Master Mix (Applied Biosystems, CA, USA) and following primers were used: CSF2RA^coop^_fwd: AAC GAG TGC TCC TGC ACC TTT AGA and CSF2RA^coop^_rev: GTT CAT CAG GTC GGC GTT GTA GA. As internal control we used the housekeeping gene *hGAPDH* (Qiagen, Hilden, Germany). Expression of pluripotency related factors *OCT4, SOX2* and *NANOG* was assessed using TaqMan Universal Master Mix II (with Uracil-N glycosylase) and gene expression assay primers for human *POU5F1 (OCT4), SOX2*, *NANOG* and *GAPDH* (Thermo Fisher Scientific, MA, USA). For all quantitative PCRs the StepOne Real Time PCR System from Applied Biosystems was used.

### Immunofluorescence

For the visualization of pluripotency related factors, iPSCs were cultured as fragments on 12–well tissue culture plates containing feeder cells to 40% confluency. Fixation of cells was performed using 4% PFA for 20 min, following permeabilisation with 0.2% Triton X-100 for 10 min on ice. BSA was used for blocking. Cells were incubated with the primary antibody at 4 °C for 1 h and the secondary antibody for 30 min at room temperature. All antibodies applied were obtained from Santa Cruz using anti-OCT4 (1:200) with a rabbit anti-mouse IgG (H + L) – Alexa546 (2 mg/ml), anti-Tra-1-60 (1:250) with a rabbit anti mouse IgG (H + L) – Alexa546 (2 mg/ml), anti-SOX2 (1:200) with a goat anti-rabbit IgG (H + L) – Alexa488 (2 mg/ml) and an anti-SSEA4 (1:250) with a goat anti-rabbit IgG (H + L)-Alexa488 (2 mg/ml).

### Microscopy

All images were examined with the inverted microscope IX71 (Olympus, Japan) equipped with the universal LUCPlanFLN objectives 4x/NA0.13, PhL, ∞/-/FN26.5; 10x/NA0.3, Ph1, ∞/-/FN26.5; 20x/NA0.45, Ph1, ∞/0-2/FN22; 40x/NA0.6, Ph2, ∞/0-2/FN22. For acquisition the Cell 2.8 software (Olympus Software Imaging Solutions GmbH) was used. All images were taken at room temperature with a resolution of 1360 × 1024 Pixel, a bit depth of 24 and a horizontal resolution 150 dpi/vertical resolution 150 dpi. The display for the individual color channels was adjusted using the Cell 2.8 software.

### Alkaline Phosphatase (AP) staining

For visualization of the alkaline phosphatase the Stemgent Alkaline Phosphatase Staining Kit (Pelo Biotech) was used and applied according to the manufacturer’s instructions. In brief, iPSCs were cultured under standard conditions to 40% confluency following their fixation and AP staining. Pictures were taken within the next two days.

### Hematopoietic differentiation of human iPSCs toward monocytes/macrophages

A previously described EB-based hematopoietic differentiation protocol was used for the generation of macrophages^32^.

### Flow cytometric analysis

The expression of different surface markers was analysed via flow cytometry using following antibodies: hCD116-PE, hCD45-PE, hCD14-PE, hCD163-APC, hCD11b-APC, hCD19-APC and isotype controls mouse-IgG1κ-PE or APC from eBioscience. Expression was analyzed using a FACSCalibur (Becton & Dickinson, Franklin Lakes, NJ, USA).

### Cytospins

Cytospins were performed with 2 × 10^4^ monocytes/macrophages with a Shandon cytocentrifuge (Thermo Scientific, Langenselbold, Germany). Following May-Grünwald-Giemsa staining, cells were allowed to dry and were covered before evaluation by bright field microscopy. Adjustments in brightness and contrast were performed using the Office Power Point software.

### IL-6 secretion

Monocytes /macrophages were terminally differentiated in 50 ng/ml human M-CSF and further cultivated in 96-well tissue culture plates for 24 h at a density of 6 × 10^4^ cells/well. After starvation for 24 h in X-Vivo 15 medium, macrophages were either left unstimulated or were stimulated with 1 µg/ml lipopolysaccharide (LPS) for another 24 h. Supernatants were collected and analysed using the human IL-6 ELISA Ready-Set-Go! Kit (Affymetrix eBioscience, CA, USA) according to the manufacturer’s instructions.

### Phagocytosis assay

For assessment of phagocytosis, pHrodo *E.coli* (#P35361 Life Technologies) were thawn freshly on ice and separated into single cells using ultrasound technology. After initial maturation of monocytes/macrophages in differentiation medium II for 5 days, cells were harvested and 2 × 10^6^ cells /500 µl RPMI (without phenolred and with 20 mmol/l Hepes) were incubated for 2 h with 25 µl pHrodo *E.coli* either at 4 °C or 37 °C, followed by incubation on ice for 10 min, before flow cytometric analysis.

### STAT5 phosphorylation assay

For the detection of pSTAT5, 2.5 × 10^5^ macrophages were seeded in a 12-well tissue culture plate in standard culture medium. Functionality of the GM-CSFRα was assessed after 24 h of starvation in X-Vivo 15 medium and subsequent culture for 1 h in X-Vivo 15 medium in absence or presence of 30 ng/ml human GM-CSF. Macrophages were harvested, fixed in 4% PFA and flow cytometric analysis was conducted using the pSTAT5-APC antibody (eBiosciences, Frankfurt am Main, Germany).

### GM-CSF uptake assay

2 × 10^5^ cells were seeded in a 12-well tissue culture plate in standard culture medium and starved for 24 h in X-Vivo 15 to be subsequently stimulated in X-vivo 15 medium containing 1 ng/ml human GM-CSF. Supernatants were collected and remaining human GM-CSF concentration was determined at time points (0 h, 2 h, 8 h, 24 h, 56 h, 104 h) using the human GM-CSF ELISA Ready-Set-Go! kit (Affymetrix eBioscience, CA, USA) according to the manufacturer’s instructions.

### Statistical analysis

For all statistical analysis One-Way ANOVA, Dunnetts post hoc test, were performed using Graph pad Prism 6.

### Data Availability

The datasets generated during and/or analysed during the current study are available from the corresponding author on reasonable request.

## Electronic supplementary material

Supplementary Material
