# Supplementary material for: TALEN-mediated functional correction of human iPSC-derived macrophages in context of hereditary pulmonary alveolar proteinosis
Source: Sci Rep. 2017 Nov 9;7:15195. doi: 10.1038/s41598-017-14566-8 (PMC5680188; doi:10.1038/s41598-017-14566-8)
Supplement: Supplementary file 1 — Supplementary Material [file 41598_2017_14566_MOESM1_ESM.pdf]

TALEN-mediated functional correction of human iPSC-derived macrophages in context of hereditary pulmonary alveolar proteinosis

Alexandra Kuhn[1,2], Mania Ackermann[1,3], Claudio Mussolino[4,5], Toni Cathomen[4,5,6], Nico Lachmann[1,3†], Thomas Moritz[1,2 *†]

[1] Institute of Experimental Hematology, Hannover Medical School, Hannover, Germany

[2] RG Reprogramming and Gene Therapy, REBIRTH Cluster of Excellence

[3]JRG Translational Hematology of Congenital Diseases, REBIRTH Cluster of Excellence

[4]Institute for Transfusion Medicine and Gene Therapy, Medical Center - University of Freiburg, Freiburg, Germany

[5]Center for Chronic Immunodeficiency, Medical Center - University of Freiburg, Freiburg, Germany

[6]Faculty of Medicine, University of Freiburg, Freiburg, Germany

Supplementary Figure 1: Genotyping of gene edited PAP patient-derived iPSCs.

Full-length agarose gels are displayed using (a) primers p1 and p3 for detection of the 5´-junction between the genomic *AAVS1* locus and the integration cassette, (b) p2 and p4 for the detection of the 3´-junction and (c) p1 and p2 for the determination of the unmodified *AAVS1* locus. Images were taken at different time points using an auto exposure time of 0.5 sec. The brightness and contrast was slightly adjusted using the Image Lab Software.

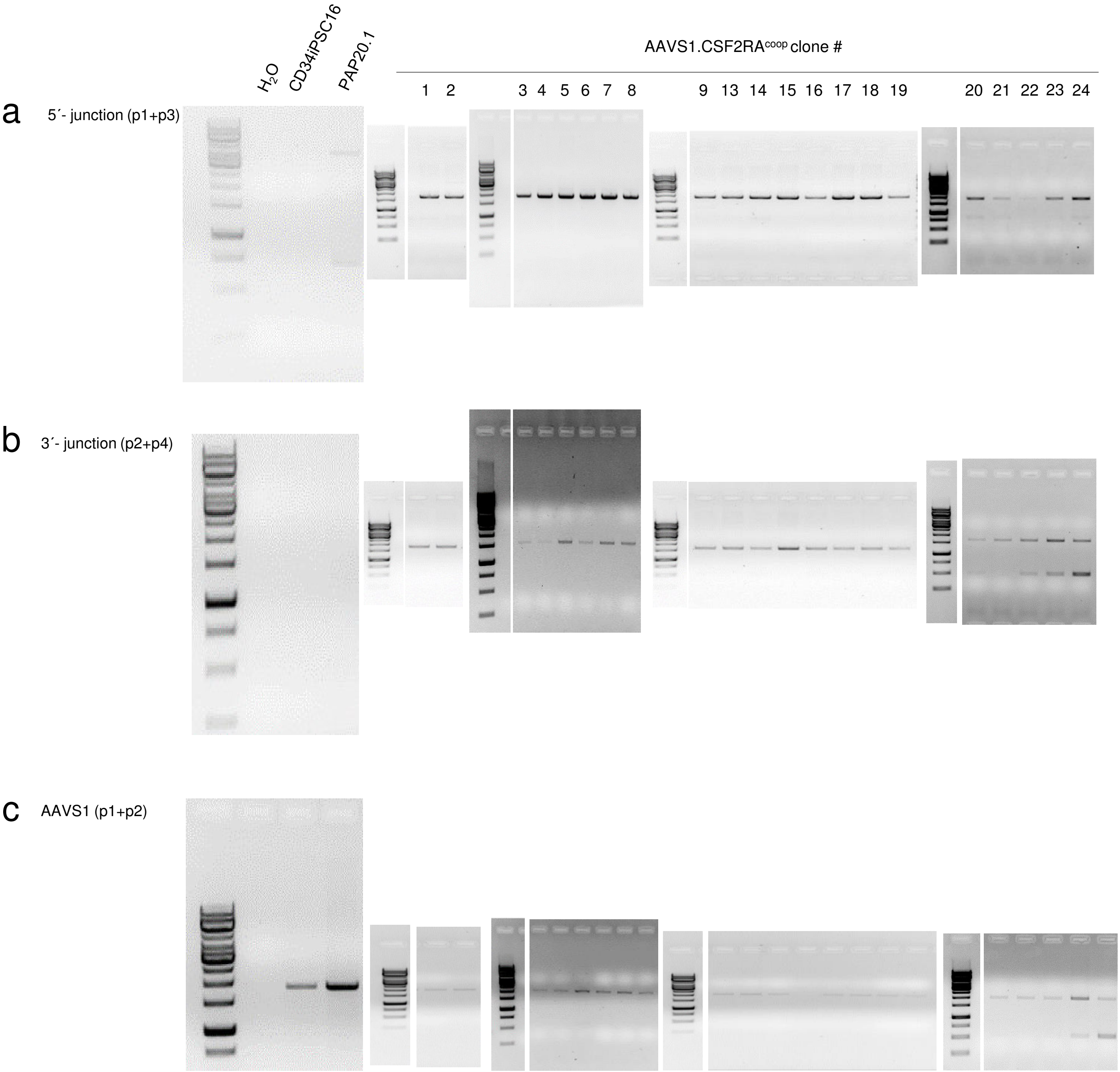
H2O
CD34iPSC16
PAP20.1
AAVS1.CSF2RAcoop clone #
1 2 3 4 5 6 7 8 9 13 14 15 16 17 18 19 20 21 22 23 24
a
5´- junction (p1+p3)
b
3´- junction (p2+p4)
c
AAVS1 (p1+p2)

TALEN-mediated functional correction of human iPSC-derived macrophages in context of hereditary pulmonary alveolar proteinosis

Alexandra Kuhn[1,2], Mania Ackermann[1,3], Claudio Mussolino[4,5], Toni Cathomen[4,5,6], Nico Lachmann[1,3†], Thomas Moritz[1,2 *†]

[1] Institute of Experimental Hematology, Hannover Medical School, Hannover, Germany

[2] RG Reprogramming and Gene Therapy, REBIRTH Cluster of Excellence

[3]JRG Translational Hematology of Congenital Diseases, REBIRTH Cluster of Excellence

[4]Institute for Transfusion Medicine and Gene Therapy, Medical Center - University of Freiburg, Freiburg, Germany

[5]Center for Chronic Immunodeficiency, Medical Center - University of Freiburg, Freiburg, Germany

[6]Faculty of Medicine, University of Freiburg, Freiburg, Germany

Supplementary Figure 2:
Representative Sanger sequencing electropherogram for 5´- and 3´-junctions between the genomic *AAVS1* site and the integration cassette.

Genomic DNA of the AAVS1.CSF2RA^{coop} #6 was isolated and sequenced to verify on-target integration within the genomic *AAVS1* locus. **(a,b)** Chromatograms for the 5´and 3' junctions between the homology arms (gray) and the integration cassette within the *AAVS1* genomic locus (white) highlight seamless homologous recombination. Ex, exon; HAL, homology arm left; T2A, self-cleaving peptide; Puro, puromycin resistance gene; CSF2RA, GM-CSF receptor alpha chain; pA, polyA site; HAR, homology arm right

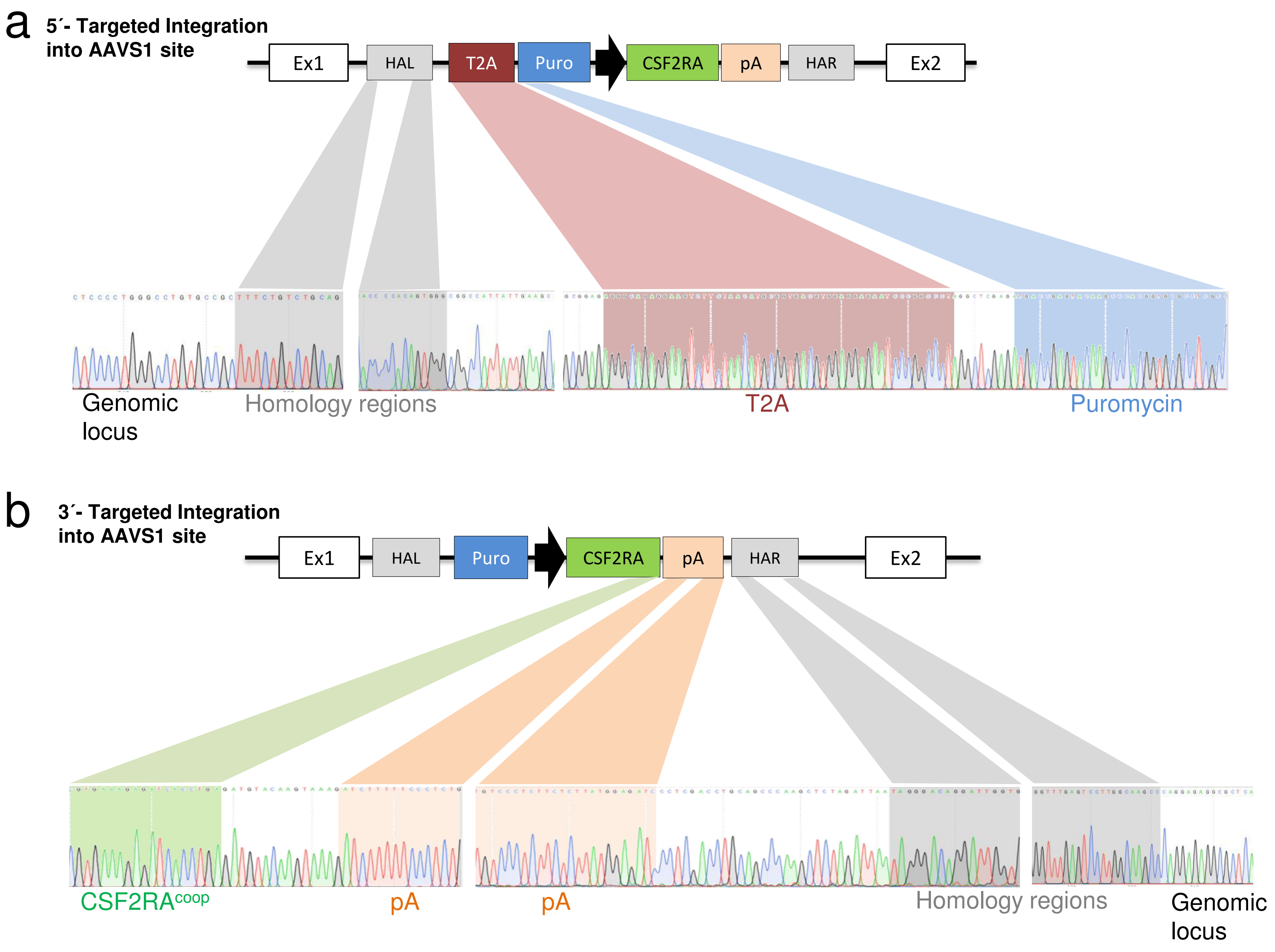

a
5´- Targeted Integration into AAVS1 site
Ex1
HAL
T2A
Puro
CSF2RA
pA
HAR
Ex2
Genomic locus
Homology regions
T2A
Puromycin
b
3´- Targeted Integration into AAVS1 site
Ex1
HAL
Puro
CSF2RA
pA
HAR
Ex2
CSF2RAcoop
pA
pA
Homology regions
Genomic locus

TALEN-mediated functional correction of human iPSC-derived macrophages in context of hereditary pulmonary alveolar proteinosis

Alexandra Kuhn[1,2], Mania Ackermann[1,3], Claudio Mussolino[4,5], Toni Cathomen[4,5,6], Nico Lachmann[1,3†], Thomas Moritz[1,2 *†]

[1] Institute of Experimental Hematology, Hannover Medical School, Hannover, Germany

[2] RG Reprogramming and Gene Therapy, REBIRTH Cluster of Excellence

[3]JRG Translational Hematology of Congenital Diseases, REBIRTH Cluster of Excellence

[4]Institute for Transfusion Medicine and Gene Therapy, Medical Center - University of Freiburg, Freiburg, Germany

[5]Center for Chronic Immunodeficiency, Medical Center - University of Freiburg, Freiburg, Germany

[6]Faculty of Medicine, University of Freiburg, Freiburg, Germany

Supplementary Figure 3:

Southern blot analysis of AAVS1.CSF2RA^{coop} iPSC clones.

a) CSF2RA^{coop} donor map. **b, c)** Schematic fragmentation of the gene-modified genome at the *AAVS1* site using the restriction enzymes SphI or NcoI: southern blot analysis using the CSF2RA^{coop} probe will result in a specific band of 7139bp (SphI) or 6320bp (NcoI) indicative of a single copy donor integration. **d, e)** Full-length southern blot image using CSF2RA^{coop}-probe (d) or AmpR-probe (e) to track the integration within the genome of six gene–modified AAVS1.CSF2RA^{coop} iPSC clones and the parental PAP20.1 iPSCs. Bands indicated with empty arrows and visible both for gene-edited clones and for PAP20.1 iPSCs, potentially representing the endogenous *CSF2RA* gene. Random integration of the donor construct (black arrow) are shown for clones #13, #18 and #23 using the CSF2RA^{coop}-probe (d) or the Ampicillin-probe (e) respectively. HAL, homology arm left; T2A, self-cleaving peptide; Puro, puromycin resistance gene; CAG, CMV early enhancer chicken beta actin promoter; CSF2RA, GM-CSF receptor alpha chain; pA, polyA site; HAR, homology arm right; AmpR, ampicillin resistance gene

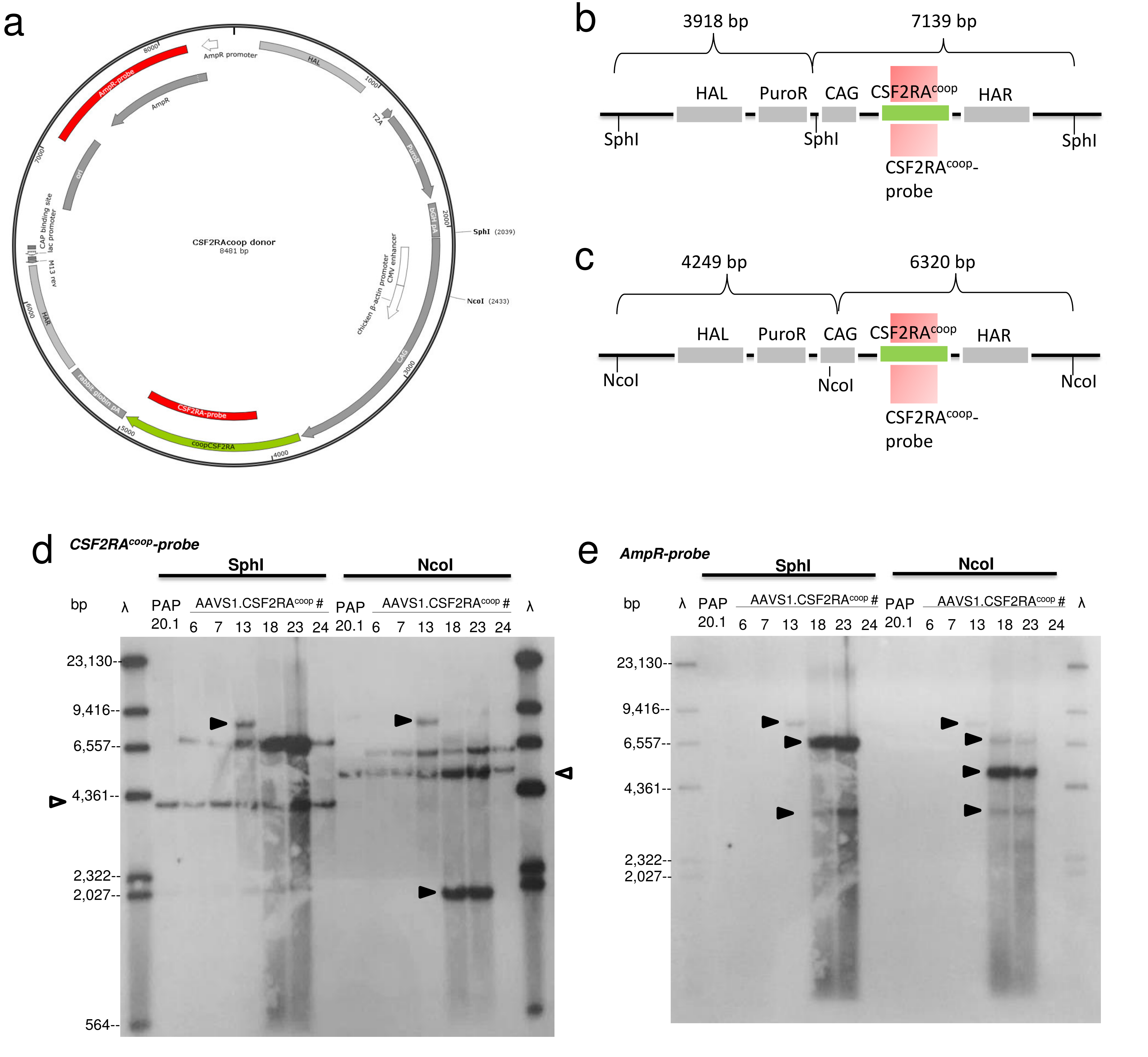
a
CSF2RAcoop donor
8481 bp
AmpR promoter
HAL
1000
T2A
PuroR
bGH pA
2000
SphI (2039)
NcoI (2433)
CMV enhancer
chicken β-actin promoter
CAG
3000
4000
coopCSF2RA
CSF2RA-probe
5000
rabbit globin pA
HAR
6000
M13 rev
lac promoter
CAP binding site
7000
ori
AmpR
AmpR-probe
8000
b
3918 bp
7139 bp
HAL
PuroR
CAG
CSF2RAcoop
HAR
SphI
SphI
SphI
CSF2RAcoop-probe
c
4249 bp
6320 bp
HAL
PuroR
CAG
CSF2RAcoop
HAR
NcoI
NcoI
NcoI
CSF2RAcoop-probe
d
CSF2RAcoop-probe
SphI
NcoI
bp
λ
PAP 20.1
AAVS1.CSF2RAcoop #
6 7 13 18 23 24
PAP 20.1
AAVS1.CSF2RAcoop #
6 7 13 18 23 24
λ
23,130
9,416
6,557
4,361
2,322
2,027
564
e
AmpR-probe
SphI
NcoI
bp
λ
PAP 20.1
AAVS1.CSF2RAcoop #
6 7 13 18 23 24
PAP 20.1
AAVS1.CSF2RAcoop #
6 7 13 18 23 24
λ
23,130
9,416
6,557
4,361
2,322
2,027
